# Sirius Scheimpflug–Placido versus ultrasound pachymetry for central corneal thickness: meta-analysis

**DOI:** 10.1186/s40662-021-00227-5

**Published:** 2021-02-18

**Authors:** Yili Jin, Colm McAlinden, Yong Sun, Daizong Wen, Yiran Wang, Jinjin Yu, Ke Feng, Benhao Song, Qinmei Wang, Shihao Chen, Jinhai Huang

**Affiliations:** 1 School of Ophthalmology and Optometry and Eye Hospital, Wenzhou Medical University, Wenzhou, Zhejiang China; 2 Department of Ophthalmology, Singleton Hospital, Swansea Bay University Health Board, Swansea, UK; 3 Shenzhen Hospital of Integrated Traditional and Western Medicine, Shenzhen, China; 4 Key Laboratory of Vision Science, Ministry of Health P.R. China, Wenzhou, Zhejiang China; 5 Eye Hospital of Wenzhou Medical University, 270 West Xueyuan Road, Wenzhou, 325027 Zhejiang China

**Keywords:** Meta-analysis, Central corneal thickness, Scheimpflug-Placido topographer, Ultrasound Pachymetry

## Abstract

**Background:**

To compare the difference in central corneal thickness (CCT) measurements in normal eyes between a rotating Scheimpflug camera combined with a Placido-disk corneal topographer (Sirius, CSO, Italy) and ultrasound pachymetry (USP).

**Methods:**

A systematic literature search was conducted for relevant studies published on PubMed, Medline, EMBASE, and the Cochrane Library and ClinicalTrials.gov from inception to August 1st, 2019. Primary outcome measures were CCT measurements between Sirius and USP. A random effects model was used to pool CCT measurements.

**Results:**

A total of twelve studies involving 862 eyes were included in this meta-analysis. The meta-analysis found CCT measurements between Sirius and USP to be statistically significantly different (*P* < 0.0001). The mean difference between Sirius and USP was −11.26 μm with a 95% confidence interval (CI) (−16.92 μm, −5.60 μm). The heterogeneity was I^2^ = 60% (*P* = 0.004).

**Conclusion:**

CCT measurements with the Sirius Scheimpflug-Placido topographer were statistically significantly lower than USP. However, it may be argued that the mean difference of 11.26 μm is not a clinically significant difference.

## Background

The precise measurement of central corneal thickness (CCT) is important in daily ophthalmic practice, in particular in the fields of corneal refractive surgery, corneal diseases and glaucoma [1–4]. Ultrasound pachymetry (USP) is still a widely used technique to measure CCT. User-friendly, low prices and understandable principle make it acceptable to clinicians. However, USP has some disadvantages. Its use requires topical anaesthesia, which may affect corneal thickness (corneal oedema). As it involves corneal contact, there is a small risk of infection. Its outcomes depend largely on the expertness of users and the probe must be aligned perpendicularly to the centre of the cornea [5]. The measurement will be at the point of contact, hence may not measure CCT accurately.

The Sirius anterior segment analysis system (CSO, Florence, Italy) utilizes a single 360-degree rotating Scheimpflug camera combined with a Placido-disk topographer of 22 rings acquiring 25 radial sections of the cornea. A blue light-emitting diode (LED) light with a wavelength of 475 nm measures 35,632 points on the anterior cornea and 30,000 points on the posterior cornea. The scanning process obtains 25 tomographic Scheimpflug images and one Placido corneal curvature in a single measurement [6, 7]. It has been reported that Sirius has high repeatability and reproducibility in measuring CCT [8–10].

Some previous studies have compared CCT measurements directly between Sirius and USP [9, 11–21]. Some have found that Sirius CCT measurements are lower than USP [11, 13–21], whereas others have found thicker measurements with the Sirius [9, 12]. The aim of this meta-analysis is to integrate all comparative studies to determine the difference of CCT measurement between Sirius and USP.

## Materials and methods

### Search methods

This meta-analysis was conducted under the guidance of the Preferred Reporting Items for Systematic Review and Meta-Analysis (PRISMA) guidelines [22]. Prospective comparative studies related to CCT measurements between Sirius and USP were identified through a systematic search by two researchers (Y.L.J. and Y.R.W.) independently using the following databases: PubMed, EMBASE, Cochrane Library and ClinicalTrail.gov. The following search terms were used: (“Ultrasonography”[Mesh] or “Ultrasonics”[Mesh] or USP or US or pachymet* or ultraso*) AND (Sirius or Scheimpflug or topograph* or tomograph*) AND (“Corneal Pachymetry”[Mesh] OR “Corneal Topography”[Mesh] OR corneal pachymet* or corneal thickness). The cut-off date was August 1st, 2019, and the language was not restricted.

### Study selection

Two independent researchers (Y.L.J. and Y.R.W.) performed the article screening; any discrepancies were resolved by focused discussion or consultation with an additional researcher (B.H.S.). Studies meeting the following inclusion criteria were included in this meta-analysis: a) Adults 18 years or older without any eye diseases or systemic diseases; b) CCT measurements were acquired both with Sirius and USP by the same clinician within 30 min; c) Compared CCT measurements directly between Sirius and USP; d) Original research. The excluded criteria were studies with incomplete data or duplicate data. The full text was obtained and reviewed with articles whose abstracts are not clear to ensure we did not leave out any article that was eligible.

### Data extraction

For all selected studies, we designed a datasheet with the following information to collect data: first author and year of publication, country of the study, sample size, number of eyes, mean and standard deviation (SD) of patient age, gender ratio, refractive error, mean and SD of CCT measured by Sirius and USP, and device manufacturer.

### Qualitative assessment

The Quality Assessment of Diagnostic Accuracy Studies (QUADAS) [23–25] scale was utilized. The primary scale comprises 14 items. As items 1, 12, 13, and 14 were not related to the present meta-analysis, they were excluded and the new scale including 10 items was used (Table 1).

**Table 1:** The refined scale of the quality assessment of diagnostic accuracy studies scale (QUADAS)

	Kara [13] 2012	Huang [9] 2013	Jorge [12] 2013	Maresca [15] 2014	Bayhan [11] 2014	Yildirim [18] 2015	Simsek [17] 2016	Pierro [16] 2016	Kumar [14] 2017	Kuddusi [19] 2017	Mustafa [20] 2018	Nesrin [21] 2018
Were selection criteria clearly described?	Y	Y	Y	Y	Y	Y	Y	Y	Y	Y	Y	Y
Is the reference standard likely to correctly classify the target condition?	Y	Y	Y	Y	Y	Y	Y	Y	Y	Y	Y	Y
Is the time period between reference standard and index test short enough to be reasonably sure that the target condition did not change between the two tests?	Y	Y	Y	Y	Y	Y	Y	Y	Y	Y	Y	Y
Did the whole sample or a random selection of the sample, receive verification using a reference standard of a diagnosis?	Y	Y	Y	Y	Y	Y	Y	Y	Y	Y	Y	Y
Did the patients receive the same reference standard regardless of the index test result?	Y	Y	Y	Y	Y	Y	Y	Y	Y	Y	Y	Y
Was the reference standard independent of the index test?	Y	Y	Y	Y	Y	Y	Y	Y	Y	Y	Y	Y
Was the execution of the index test described in sufficient detail to permit replication of the test?	Y	Y	Y	Y	Y	Y	Y	Y	Y	Y	Y	Y
Was the execution of the reference standard described in sufficient detail to permit its replication?	Y	Y	Y	Y	Y	Y	Y	Y	Y	Y	Y	Y
Were the index test results interpreted without knowledge of the results of the reference standard?	Y	Y	Y	Y	Y	Y	Y	Y	Y	Y	Y	Y
Were the reference standard results interpreted without knowledge of the results of the index test?	U	Y	Y	Y	Y	Y	U	Y	U	Y	Y	Y

*Y* = yes, *N =* no, *U* = unclear

### Outcomes

The measurement results of the CCT by Sirius and USP were compared in normal eyes.

### Statistical analysis

Statistical analysis was performed with Review Manager (version 5.3, Cochrane Collaboration, Oxford, UK) statistical analysis software. As CCT is a continuous outcome, weighted mean differences (WMD) with 95% confidence intervals (CI) were calculated and the difference was defined as CCT measurement of Sirius minus USP. The fixed effects model was used to analyse the data initially. Heterogeneity was assessed by the I^2^ parameter. I^2^ is a statistic for quantifying inconsistency and is calculated as follows: I^2^ = (Q − df/Q) × 100%, where Q is the Chi-squared statistic and df is the degrees of freedom. In other words, I^2^ quantifies the percentage variability due to heterogeneity rather than by chance [26]. If the I^2^ was > 50%, the random effects model was used to pool the data for the relatively higher variance in clinical characteristics and sample sizes. A *P*-value of less than 0.05 was considered to be statistically significant. Sensitivity analysis was performed by omitting one study at a time and re-evaluating the effect of different statistical models (fixed effects model or random effects model). Ultrasonic velocity was likely to be one of the sources of heterogeneity, so we analysed the heterogeneity based on whether the ultrasonic velocity was reported and the reported speed. Subgroup analysis was carried out based on the different races included in studies. A funnel plot is a scatter plot to assess for publication bias. The x-axis of the funnel plot signifies the mean result (e.g., mean difference, odds ratio, risk ratio) whereas the y-axis signifies the index of precision (e.g., standard error) or sample size. One would expect to observe an even scattering around the true result, however when significant publication bias exists, an asymmetry in the scatter is observed [27].

## Results

**Fig. 1:**
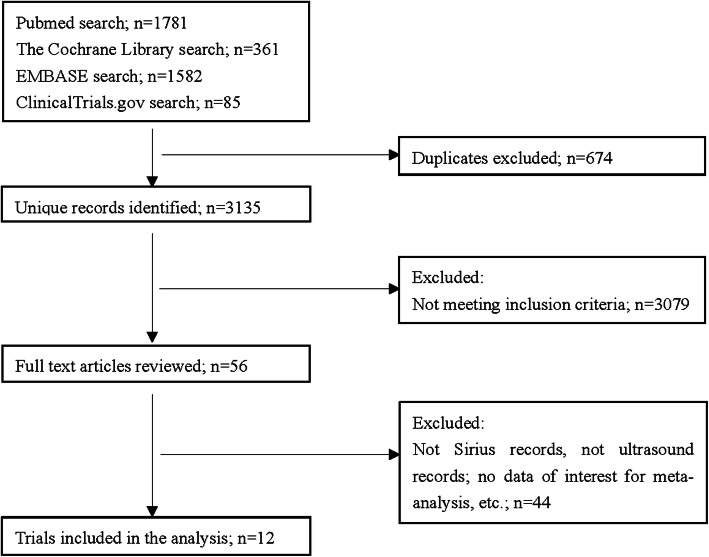
Selection flowchart of the studies included in the meta-analysis

The systematic search flowchart is showed in Fig. 1. After duplication was excluded, 3135 articles conformed to our terms. Three thousand seventy-nine articles were excluded through screening their title and abstract. The full text of the remaining 56 articles were obtained and 44 were excluded since 39 did not record CCT measurements for Sirius or USP, two studies compared patients with keratoconus, two studies compared patients following laser in situ keratomileusis (LASIK), one study involved patients with acne vulgaris. There were 12 final studies that met our criteria [11]. The enrolled studies and basic data are presented in Table 2. A total of 862 eyes were included in the meta-analysis.

**Table 2:** Basic study information

Author	Year	Country	Patients/Eyes	Mean age ± SD (years)	Gender ratio (M/F)	Device manufacturer	
						Sirius	Ultrasound
Kara et al. [13]	2012	Turkey	30/30	28.0 ± 10.0	14/16	Sirius (CSO, Florence, Italy)	DGH-550 (DGH Technology Inc., Exton, PA)
Huang et al. [9]	2013	China	43/43	27.5 ± 7.6	16/27	Sirius (CSO, Florence, Italy)	SP-3000 (Tomey Corp.)
Jorge et al. [12]	2013	Portugal	50/50	36.7 ± 4.8	28/22	Sirius (CSO, Florence, Italy)	SP100 (Tomey, Nagoya, Japan)
Bayhan et al. [11]	2014	Turkey	50/50	29.8 ± 5.0	23/27	Sirius (CSO, Florence, Italy)	(Pacline, Optikon)
Maresca et al. [15]	2014	Italy	35/35	25.9 ± 6.7	10/25	Sirius (CSO, Florence, Italy)	P-1 (Takagi Seiko Co., Ltd., Nagano, Japan)
Yildirim et al. [18]	2015	Turkey	34/34	23.2 ± 7.6	16/18	Sirius (CSO, Florence, Italy)	DGH-550 (DGH Technology Inc., Exton, PA).
Pierro et al. [16]	2016	Italy	28/28	44.0 ± 10.6	8/20	Sirius (CSO, Florence, Italy)	Pachmate DGH55 (DGH Instruments Inc., Exton, PA)
Simsek et al. [17]	2016	Turkey	128/256	33.2 ± 13.0	84/44	Sirius (CSO, Florence, Italy)	OcuScan RxP Ophthalmic Ultrasound System (Alcon Laboratories)
Kumar et al. [14]	2017	India	46/46	24.1 ± 5.0	24/22	Sirius (CSO, Florence, Italy)	SP-3000 (Tomey Corp.)
Kuddusi et al. [19]	2017	Turkey	76/76	38.6 ± 12.5	33/43	Sirius (CSO, Florence, Italy)	Echoscan US-500
Mustafa et al. [20]	2018	Turkey	32/64	23.8	25/7	Sirius (CSO, Florence, Italy)	Aviso A/B (Quantel Medical, France)
Nesrin et al. [21]	2018	Turkey	150/150	42.5 ± 17.0	67/83	Sirius (CSO, Florence, Italy)	iPac ultrasonic pachymeter

*SD* = standard deviation, *CCT* = central corneal thickness

**Fig. 2:**
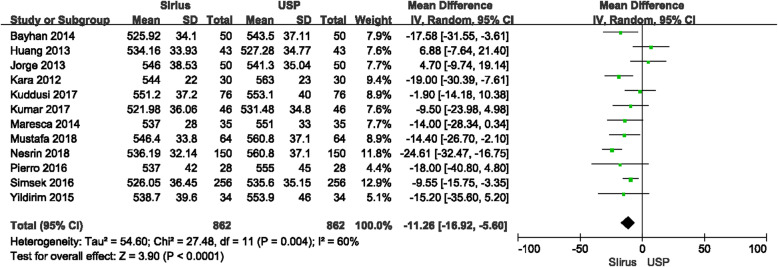
Comparison of central corneal thickness (CCT) measurements with the Sirius and ultrasound pachymetry (USP). df = degree of freedom; I^2^ = extent of inconsistency; Z = overall effect

**Fig. 3:**
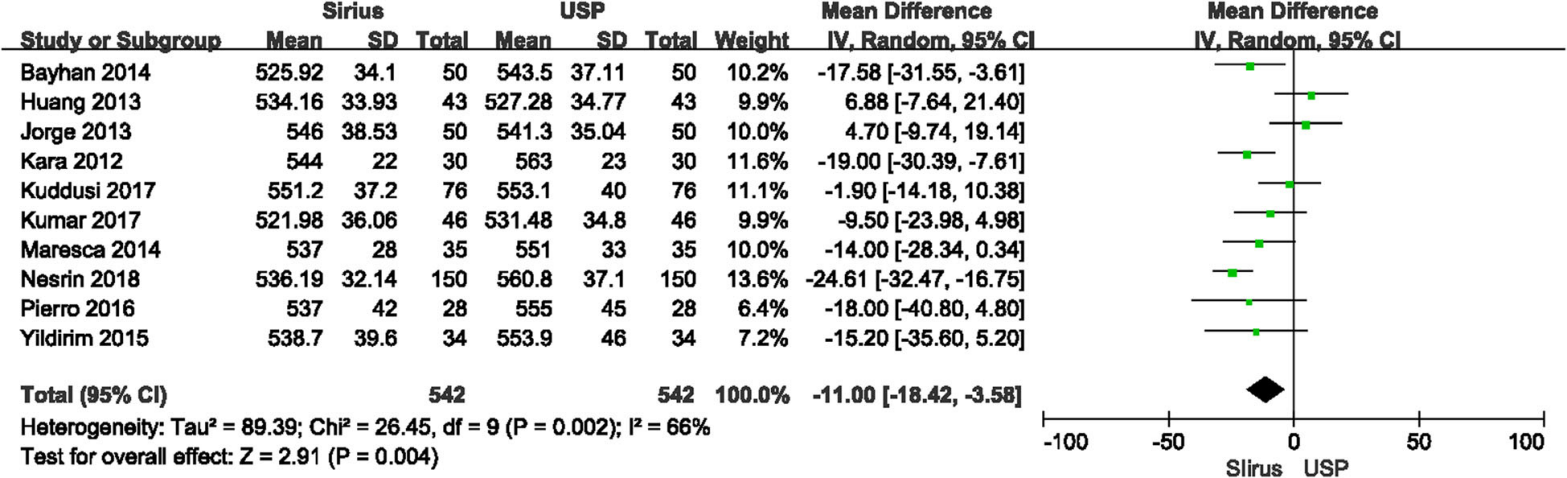
Comparison of central corneal thickness (CCT) measurements with the Sirius and ultrasound pachymetry (USP) (excluded both-eyes studies). df = degree of freedom; I^2^ = extent of inconsistency; Z = overall effect

**Fig. 4:**
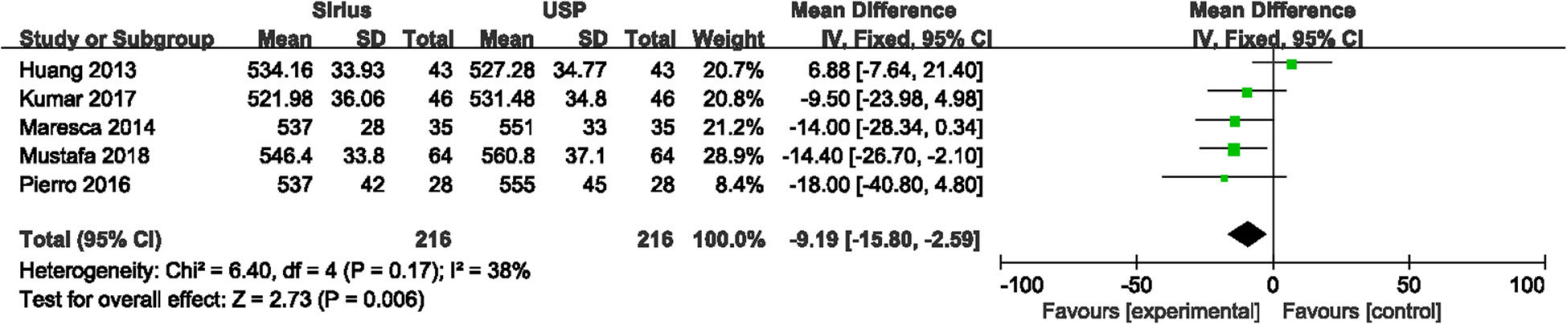
Comparison of central corneal thickness (CCT) measurements with the Sirius and ultrasound pachymetry (USP) that reported ultrasonic speed. df = degree of freedom; I^2^ = extent of inconsistency; Z = overall effect

CCT measurements between Sirius and USP were statistically significantly different (*P* < 0.0001), with the forest plot displayed in Fig. 2. The mean difference between Sirius and USP was −11.26 μm with the 95% CI (−16.92 μm, −5.60 μm). The heterogeneity (I^2^) was 60% (*P* = 0.004). Since two studies [17, 20] included both eyes, in order to rule out the impact of the similarity of the both eyes on the meta-analysis, we excluded these two studies and performed another analysis. The results showed that in a total of 542 eyes, the mean difference between Sirius and USP was −11.00 μm with 95% CI (−18.42 μm, −3.58 μm), and the I^2^ was 66% (Fig. 3). There was no significant difference with the overall group. These studies were carefully examined to find the source of the heterogeneity. When using a fixed effects model, the mean difference was −11.98 μm with 95% CI of −15.22 μm to −8.73 μm (*P* < 0.00001) which is no different than the random effects model. The primary heterogeneity was high, so a sensitivity analysis was performed by excluding each study in turn. When the study by Gokcinar et al. [21] was excluded, the heterogeneity decreased from 60% to 37%. Reviewing this article carefully, there were slight differences in the inclusion criteria. The refractive error was in a range of −5.00 D to +5.00 D; the corrected distance visual acuity (CDVA) was better than 20/25 (not 20/20 akin to other studies); and contact lens wear was permitted up to 72 h prior to measurement, whereas other studies have reported contact lens wear was ceased a least month prior to measurements. Three other studies [9, 12, 14] with these different inclusion criteria were excluded and the heterogeneity decreased to 0%, the mean difference was −11.95 μm with the 95% CI were −15.99 μm to −7.91 μm (*P* < 0.00001) with the fixed effects model. The difference which excluded three articles has no difference with the primary group. In addition, we extracted studies which reported the ultrasonic velocity of USP were extracted. Five studies with 216 eyes reported ultrasonic sound velocity, all were 1640 m/s. The difference between Sirius and USP was −9.19 μm with 95% CI from −15.80 μm to 2.59 μm (*P* = 0.006), I^2^ = 38% (Fig. 4), suggesting that the CCT measured by Sirius was lower than that of USP, which is consistent with the primary analysis.

### Subgroup analysis

**Fig. 5:**
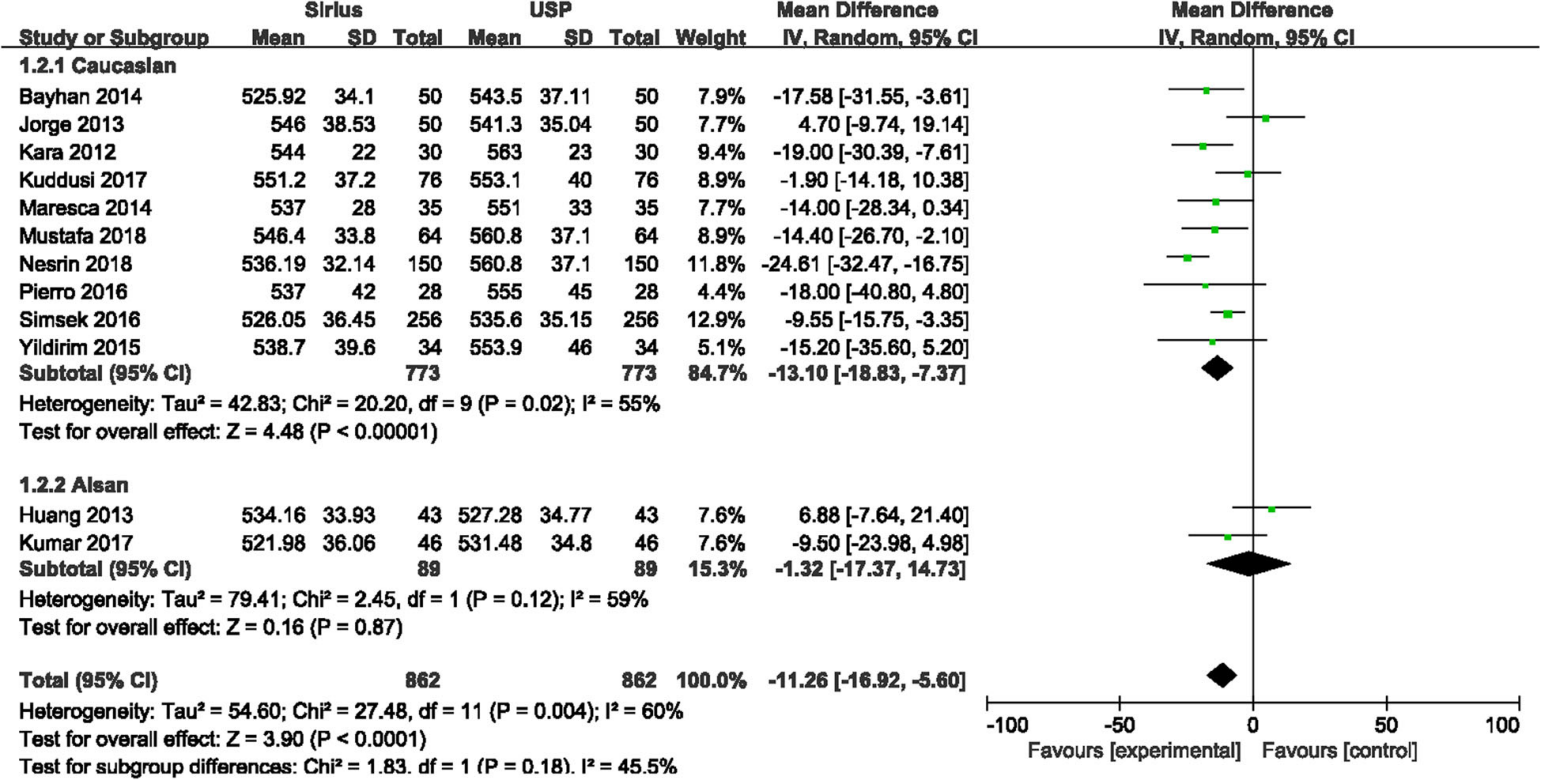
Subgroup analysis (Caucasian or Asian) of central corneal thickness (CCT) measurements with the Sirius and ultrasound pachymetry (USP).df = degree of freedom; I^2^ = extent of inconsistency; Z = overall effect

According to the different races of the subjects included in these studies, they were divided into two groups of Caucasians [11–13, 15–21] and Asians [14, 28] for analysis (Fig. 5). It was found that in Caucasians, the mean difference of CCT measurements between Sirius and USP was −13.10 μm with 95% CI from −18.83 μm to −7.37 μm (*P* < 0.0001), which was not significantly different to the overall group; while the mean difference in the Asian group was −1.32 μm with 95% CI from −17.37 μm to 14.73 μm (*P* = 0.87).

### Publication Bias

**Fig. 6:**
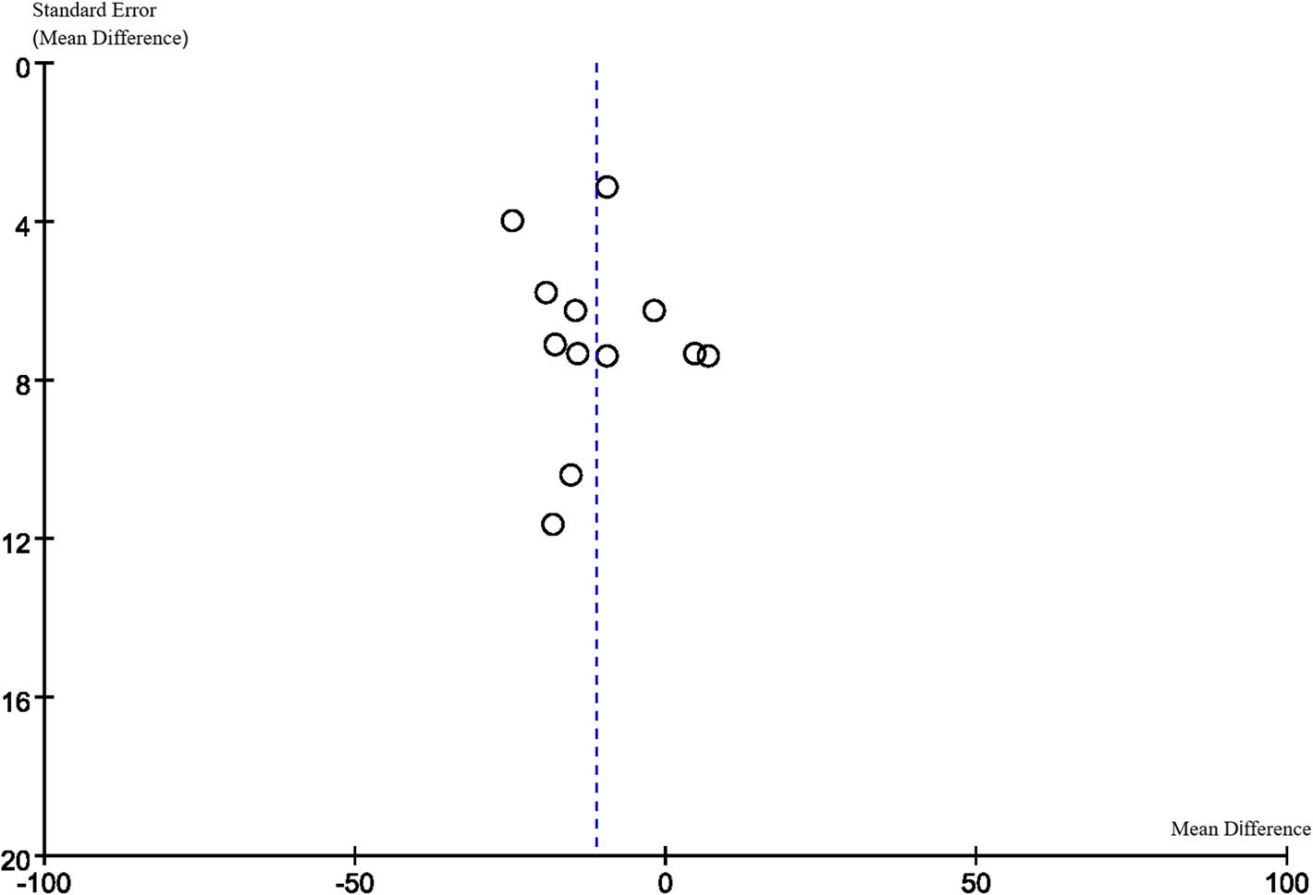
Funnel plot of overall group

**Fig. 7:**
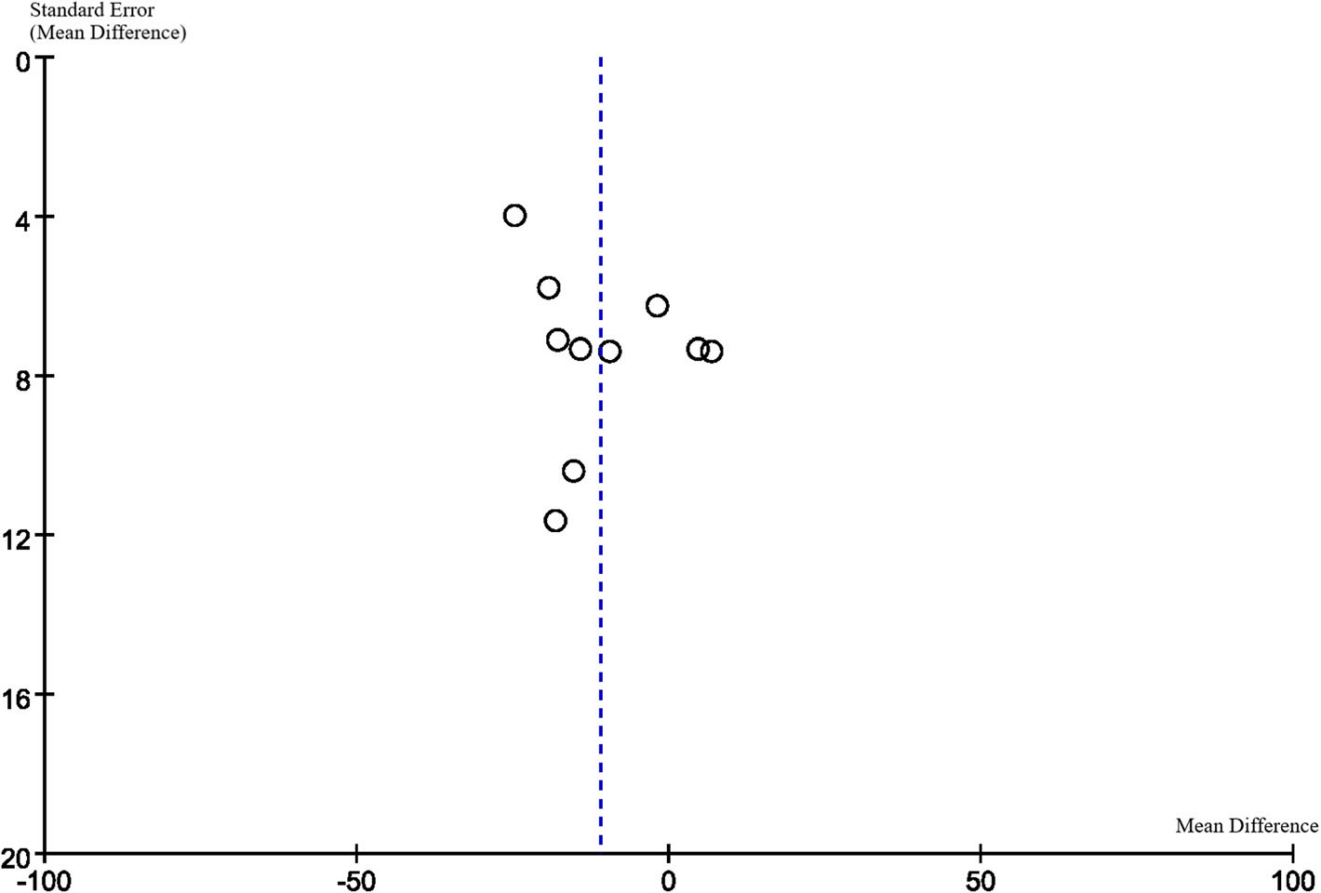
Funnel plot of excluded both-eyes studies

**Fig. 8:**
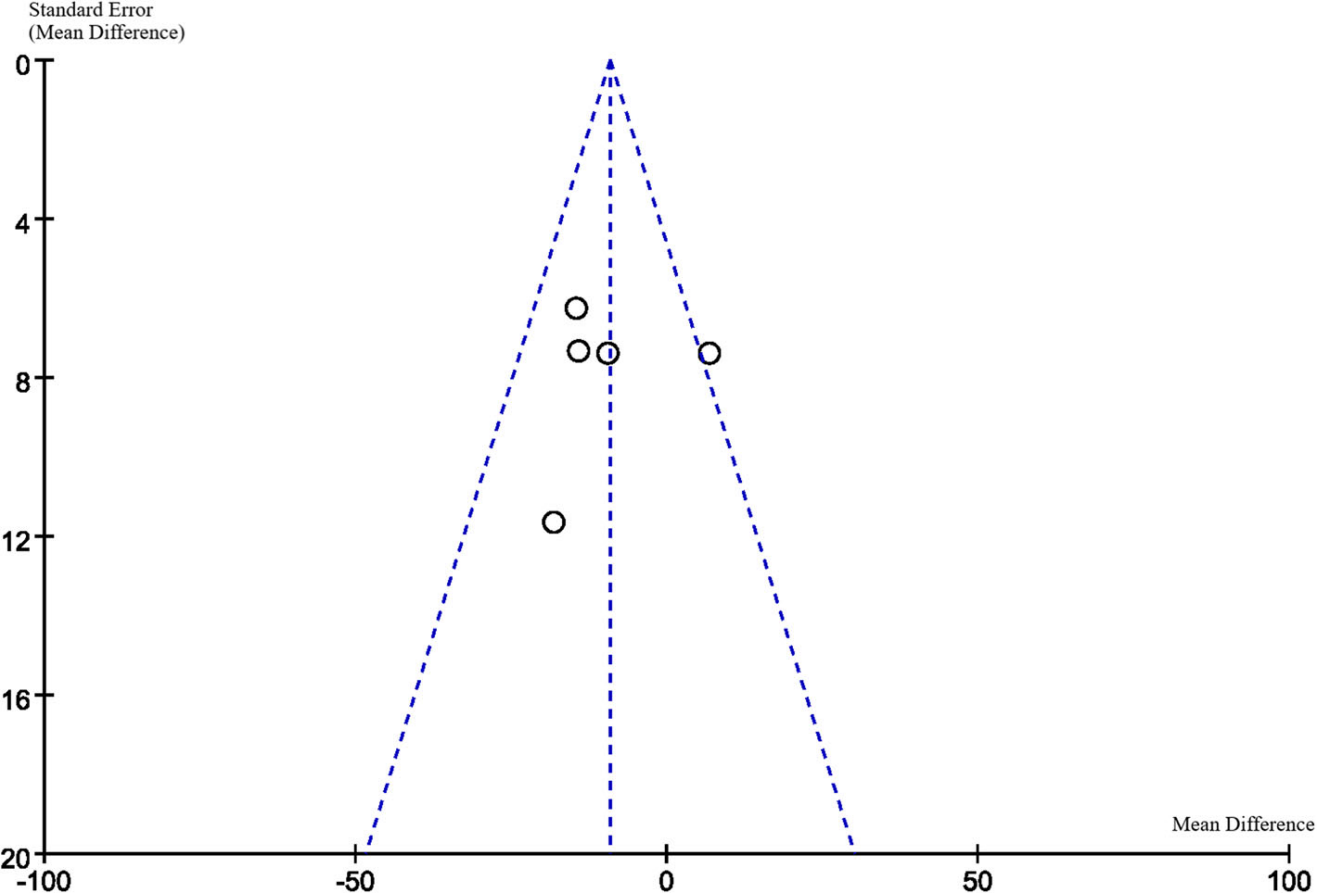
Funnel plot of reported ultrasonic speed studies

**Fig. 9:**
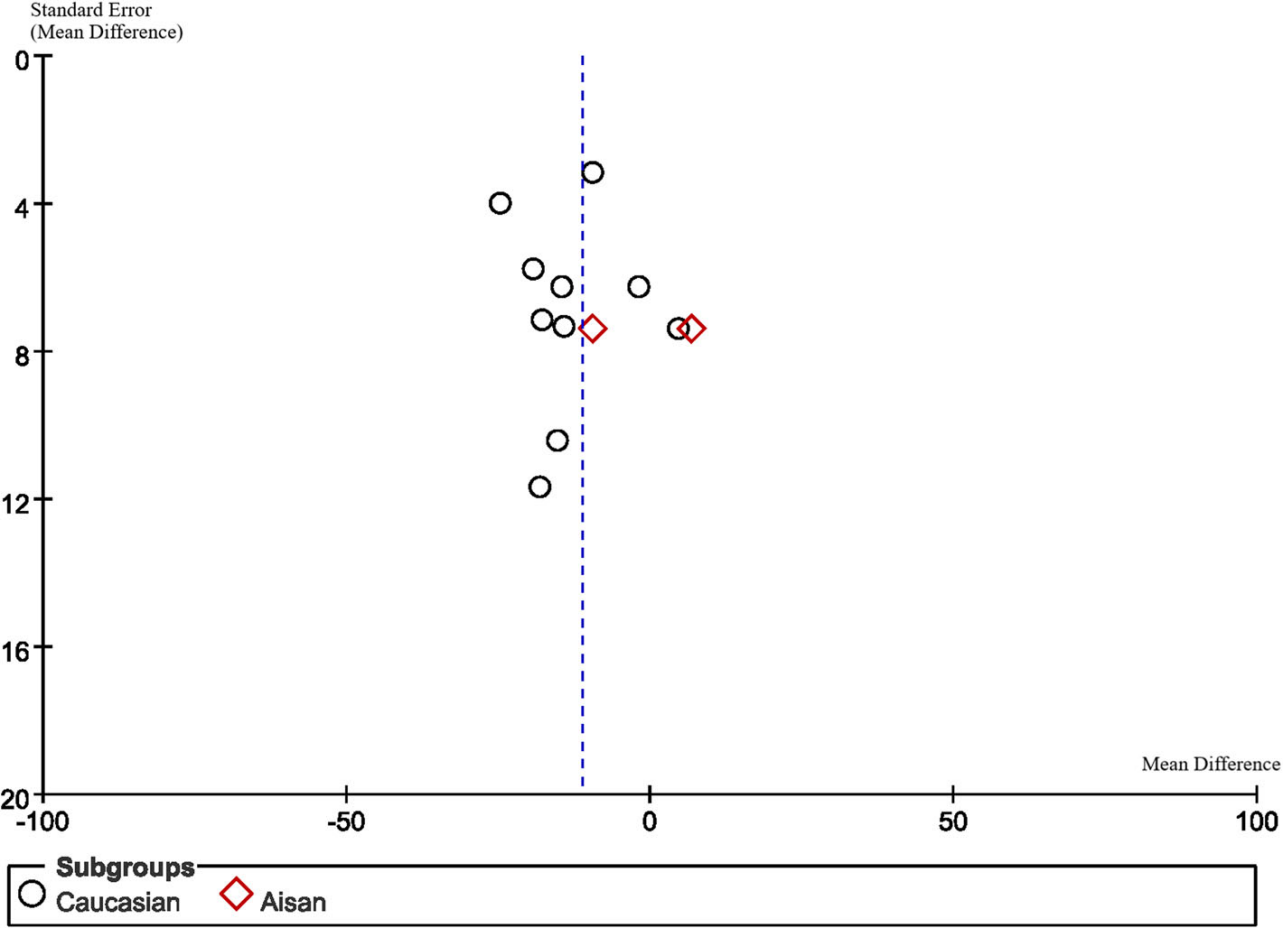
Funnel plot of subgroup (Caucasian or Asian)

Funnel plots are displayed in Figs. 6, 7, 8 and 9. The funnel plots are symmetrical except for the Asian group, suggesting this subgroup may have publication bias.

## Discussion

To the best of our knowledge, this study is the first meta-analysis to compare the Sirius with USP in terms of CCT measurements in normal healthy corneas. Most of the included studies have sample sizes ranging from 30 to 60 eyes. Our meta-analysis analysed 862 eyes by combining results of the included studies, which provides great statistical power than that of each study alone [29].

Our study suggests that, in normal healthy eyes, CCT measured with the Sirius were statistically lower than that measured with USP; the mean difference was −11.26 μm. A previous study [9] found that the 95% LoA with CCT measurements between Sirius and USP was −6.39 μm to 20.14 μm indicating an acceptable level of agreement in normal healthy eyes. Bayhan et al. [11] found that the mean difference of CCT measurement between Sirius and USP was −17.58 μm with the 95% CI (−19.89 μm to −15.27 μm), which was consistent with the present meta-analysis. The mean difference in the study by Dogan et al. [20] was −14.4 μm, again, similar to the present study. Similar results were also obtained by Pierro et al. [16] with a mean difference of −18 μm. Teberik et al. [19] found a mean difference of only −1.9 μm, in which the Sirius only marginally underestimated the CCT in comparison to USP. Gao et al. [30] reported that eye drops could substantially increase corneal thickness by more than 20 μm in 63% of patients. Topical anaesthesia can interfere with the integrity of the tear film, causing corneal oedema, making the ultrasound speed set and the exact sound speed in the corneal tissue inconsistent. As an optical biometer, Sirius does not have this issue as long as the clinician instructs the patient to blink and keep the tear film intact. In addition, the accuracy of USP depends largely on the experience of the observer; the probe must perpendicularly align to the centre of the cornea. Supposing the probe is not perpendicular, the ultrasound wave would enter the cornea tilted and could increase the value of corneal thickness. In addition, differences may occur due to incorrectly locating the probe at the exact corneal centre. The Sirius selects the corneal vertex normal as the reference centre. USP identifies the posterior reflection point located between Descemet membrane and the anterior chamber. Choosing the posterior reflection point closer to the anterior chamber may result in an overestimation [28].

Other Scheimpflug based devices (namely Pentacam and Galilei) were not included in this meta-analysis as a previous meta-analysis has been published comparing the Pentacam to USP, and only three articles compared the Galilei to USP. Wu et al. [29] conducted a meta-analysis comparing the Pentacam and USP (19 studies, 1908 eyes). It was reported that the difference between the Pentacam and USP for CCT measurement was 1.47 μm (95% CI −2.32 to 5.27 μm, *P* = 0.45). Subsequent studies reported a CCT difference between the two between 0.05 μm and 4.7 μm [31–33]. Menassa et al. [34] evaluated the difference between the Galilei and USP, with a reported difference of 6.8 ± 9.8 μm for CCT. Subsequent similar studies have reported differences between 0.55 μm and 0.7 μm [35, 36]. However, the results from a study Hosseini et al., reported a difference of 11.96 μm between the two [37].

After reading the full text of articles carefully, we found that the difference in the refractive error of the sample may be one of the reasons for the heterogeneity. In the study by Huang et al. [9], the refractive error range was −0.50 D to −12.0 D; the refractive error of the population included in Gokcinar et al. [21] was between −5.00 D and +5.00 D. In the study by Kumar et al. [14], it was between −6.00 D and +6.00 D. Other studies included samples with refractive errors between −2.00 D and +2.00 D. Five studies did not report refractive error. Pedersen et al. [38], using an optical low-coherence reflectometry pachymeter, reported thinner CCT in patients with high myopia (> −6.00 D) compared to those with emmetropia; 527.7 μm versus 538.6 μm, respectively. Chang et al., Wang et al. and Kunert et al. [39–41] reported differing CCT across patients from different ethnicities. Many studies [42–44] have shown that wearing contact lenses will significantly change CCT, short-term wear makes CCT thicker, long-term wear makes CCT thinner and increases corneal surface irregularities. A period of approximately 1 month is needed for CCT to return to normal. Since we are unable to know the number of samples and the length of time of wearing the contact lens, the heterogeneity of including such samples is unpredictable. We found that the I^2^ decreased to 38% when analysing specifically studies which reported ultrasonic velocity. The ultrasonic velocity was 1640 m/s in all these studies and the results had no difference with the primary group, and we could not determine its specific effect because other studies have not reported the exact ultrasonic velocity. We are still unclear about the causes of heterogeneity in CDVA differences [45]. A possible explanation is that subjects with decreased CDVA have potential eye diseases that can alter CCT, which increases the heterogeneity of the included samples. This may partly explain the high heterogeneity in the meta-analysis and the rapid decline in heterogeneity after exclusion of these studies.

CCT is an important step in preoperative screening and preoperative planning for corneal refractive surgery. Studies have shown that in order to avoid corneal ectasia, the absolute CCT needs to be greater than 450 μm or a residual stromal bed thickness after ablation of > 250 μm [46]. A previous study [47] investigated subjects who wanted to undergo refractive surgery and found that 55.1% of the subjects were unable to undergo refractive surgery because of insufficient corneal thickness. As the mean difference found in our meta-analysis between the two techniques was 11.26 μm, this is lower than the diurnal variation and hence we assert this difference, although statistically significant, is not clinically significant.

A large number of studies [48–50] have shown that the IOP is highly correlated with CCT. Foster et al.^51^ used an optical corneal pachymeter and a Goldmann applanation tonometer to analyse the correlation between CCT and IOP in the Mongolian population. It was found that for every 10 μm increase in CCT, the IOP only increased by 0.12 mmHg to 0.31 mmHg, but inter-individual difference may produce an IOP difference of 2.3 mmHg to 3.1 mmHg. Wolfs et al. [49] reported that each 10 μm increase in CCT caused an increase in IOP of 0.09 to 0.28 mmHg. Doughty et al. [50] indicated that a 10% change in CCT would only cause a difference in the IOP measurement with applanation tonometry of 1.1 ± 0.6 mmHg, which is not clinically significant. The current meta-analysis shows that the healthy human CCT measured by Sirius is 11.26 μm lower than the USP, which is much smaller than 10% of the thickness of a normal cornea. In view of the clinically common non-contact tonometer instead of applanation tonometer, the IOP correction error caused by the difference in CCT measurement between the two does not cause clinical misunderstanding of IOP.

There are several limitations in the current meta-analysis. First, we only included normal healthy adult populations and did not evaluate a variety of more complex situations such as children, cataracts, and keratoconus. This was mainly due to the lack of studies outside normal healthy eyes. Second, we did not include unpublished articles, such as posters or abstracts from conferences. Third, various USP devices were used in the studies included in this meta-analysis, although we assessed heterogeneity for studies reporting ultrasonic velocity. These USP devices are likely to vary in their precision (repeatability and reproducibility) which can have a knock-on effect on the agreement with the Sirius.

## Conclusions

In summary, the present meta-analysis suggests that there are statistically significant differences in the measurement of CCT between the Sirius Scheimpflug-Placido topographer and USP in normal adult eyes and the value of Sirius is lower than the value of USP. This difference is small and below normal diurnal variation and is not considered clinically significant.

## Data Availability

The datasets supporting the conclusions of this article are included within the article and its additional file.
